# Drosophila Embryonic Cell-Cycle Mutants

**DOI:** 10.1534/g3.113.007880

**Published:** 2013-10-01

**Authors:** Yingdee Unhavaithaya, Eugenia A. Park, Irena Royzman, Terry L. Orr-Weaver

**Affiliations:** * Whitehead Institute, Massachusetts Institute of Technology, Cambridge, Massachusetts 02142; † Department of Biology, Massachusetts Institute of Technology, Cambridge, Massachusetts 02142

**Keywords:** *Drosophila melanogaster*, cell cycle, *pav*, *tum*, *cyclin E*, *cal1*, *spc105R*

## Abstract

Nearly all cell division mutants in Drosophila were recovered in late larval/pupal lethal screens, with less than 10 embryonic lethal mutants identified, because larval development occurs without a requirement for cell division. Only cells in the nervous system and the imaginal cells that generate the adult body divide during larval stages, with larval tissues growing by increasing ploidy rather than cell number. Thus, most mutants perturbing mitosis or the cell cycle do not manifest a phenotype until the adult body differentiates in late larval and pupal stages. To identify cell-cycle components whose maternal pools are depleted in embryogenesis or that have specific functions in embryogenesis, we screened for mutants defective in cell division during embryogenesis. Five new alleles of *Cyclin E* were recovered, ranging from a missense mutation that is viable to stop codons causing embryonic lethality. These permitted us to investigate the requirements for Cyclin E function in neuroblast cell fate determination, a role previously shown for a null *Cyclin E* allele. The mutations causing truncation of the protein affect cell fate of the NB6-4 neuroblast, whereas the weak missense mutation has no effect. We identified mutations in the *pavarotti* (*pav*) and *tumbleweed* (*tum*) genes needed for cytokinesis by a phenotype of large and multinucleate cells in the embryonic epidermis and nervous system. Other mutations affecting the centromere protein CAL1 and the kinetochore protein Spc105R caused mitotic defects in the nervous system.

As organisms progress from a single cell, fertilized embryo to a multicellular adult, cell division must be coordinated with developmental cues. Identification of mutants defective in cell division provides an entry point for elucidating the regulatory signals between division and differentiation. Such mutants also can reveal unique cell-cycle control used to achieve particular developmental strategies.

Drosophila development requires that two body plans be constructed: a larval body and an adult body, the latter built from differentiating imaginal cells during pupation as larval tissues are histolyzed. Embryogenesis produces both the larval body and the imaginal cells. In embryogenesis 13 rapid divisions occur in a nuclear syncytium under maternal control (Figure 1). After cellularization and the onset of zygotic gene expression, there are three additional mitotic divisions in most embryonic cells, the postblastoderm divisions. At this developmental transition, the cells of the epidermis exit the cell cycle, cells in larval tissues enter the endocycle (an S-G cycle that increases ploidy), whereas mitotic divisions continue in the nervous system. During larval development, mitotic divisions occur only in the nervous system and in diploid imaginal tissues.

**Figure 1:**
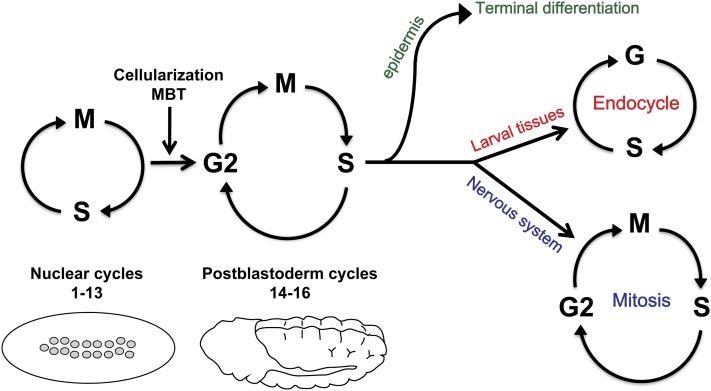
Schematic of cell cycle changes in Drosophila embryogenesis. There are 13 rapid S-M divisions in the Drosophila embryo under maternal control, followed by three postblastoderm divisions. These latter divisions follow the onset of zygotic transcription, are controlled by zygotic expression of the *string* cdc25 phosphatase, and are slowed by the presence of a G2 phase in the cell cycle. After these divisions, cells in the epidermis exit the cell cycle while cells in differentiating larval organs add a G1 phase and enter the endocycle. Subsequent G1-S transitions are marked by transcriptional induction of genes required for S phase, the basis for our genetic screen by *in situ* hybridization of *PCNA*. Mitotic divisions continue in the developing nervous system. (MBT, Midblastula transition, the transition from maternal to zygotic control).

Nearly all Drosophila cell-cycle mutants recovered from forward genetic screens were identified by a phenotype of late larval/pupal lethality due to a failure of cell division in imaginal tissues (Gatti and Baker 1989; Gatti and Goldberg 1991). Maternal stockpiles of essential mitotic proteins were presumed to accommodate the embryonic mitotic divisions in these mutants. Because larval tissues grow by increasing ploidy, and thus cell size, cell division defects in the imaginal tissues are not manifested until the imaginal cells begin to differentiate to form the adult body.

Fewer than 10 cell-cycle mutants with embryonic lethality have been identified. Most of these are the consequence of mutations in genes encoding proteins that are turned over in the cell cycle, thus eliminating maternal pools. Examples of these include the Cyclins A and E, the Cdc20 homolog Fizzy, the Securin Pimples, and the Separase subunit Three Rows (D’Andrea *et al.* 1993; Knoblich and Lehner 1993; Knoblich *et al.* 1994; Leismann *et al.* 2000; Sigrist *et al.* 1995; Stratmann and Lehner 1996). Other mutants have an embryonic phenotype because their protein products are required to alter the cell cycle or cell division in response to developmental cues, such as the addition of cytokinesis after cycle 13 (Hime and Saint 1992; Lehner 1992), the cessation of mitosis by the Cdh1 homolog Fizzy Related (Sigrist and Lehner 1997), or action of the Cdk inhibitor Dacapo to add a G1 cell cycle phase (de Nooij *et al.* 1996; Lane *et al.* 1996). A mutation in a subunit of the Condensin complex causes mitotic defects in the nervous system late in embryogenesis, possibly because the maternal stockpiles become depleted by continued mitosis in the nervous system (Bhat *et al.* 1996).

We reasoned that identifying additional Drosophila cell-cycle mutants with embryonic defects could identify proteins that are turned over in the cell cycle or regulators with specific developmental roles in embryogenesis.

## Methods and Materials

### Screen design

**Figure 2:**
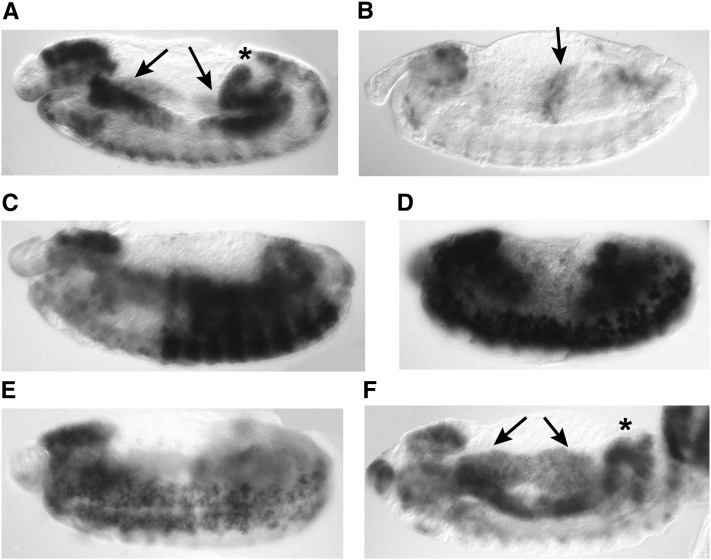
*PCNA in situ* hybridization patterns. (A) A stage 13 embryo has *PCNA* transcripts in the nervous system, the anterior and posterior midgut (arrows), and in the hindgut and malpighian tubules (asterisk). All of these tissues except the nervous system are endocycling. (B) At stage 15 *PCNA* transcripts are detected in the midgut as it enters its second round of the endocycle (arrow), but transcripts have been down-regulated in the tissues that were in endocycle S phase earlier in embryogenesis. (C) The pattern of *Ubx*-driven *lacZ* expression used to identify heterozygous embryos in the screen. The *PCNA in situ* pattern can be seen in the nervous system and internal organs. (D) The mutants that arrested in the postblastoderm divisions were diagnosed by a reduced number of large cells staining with *PCNA*, as in the *pimples* mutant shown here. (E) The *PCNA in situ* pattern revealed large cells in the CNS. (F) Mutations in *Cyclin E* caused persistent *PCNA* expression, as evidenced by this stage 15 embryo with *PCNA* transcripts still present in the anterior and posterior midguts (arrows) and hindgut (asterisk). The original *32a* mutant is shown in (E) and (F); this mutant carried a mutation in *tum* responsible for the large nuclei in the CNS as well as a mutation in *Cyclin E*. In all the panels anterior is on the left and dorsal on the top.

Our laboratory conducted a screen for regulators of the G1-S transition of the cell cycle (Royzman *et al.* 1997). In embryogenesis, the first G1-S transition occurs after division cycle 16 (Figure 1), is under developmental control, and is stereotypically patterned (Duronio and O’Farrell 1994; Smith and Orr-Weaver 1991). This transition is driven by transcriptional activation of multiple genes required for S phase, permitting us to use *in situ* hybridization to the *PCNA* gene to score for defects in the G1-S transition in embryos from mutant lines (Figure 2) (Duronio and O’Farrell 1994). Because we visually examined the *in situ* pattern in individual embryos, we identified embryos in which the postblastoderm divisions were not completed and thus had reduced cell number, embryos with large cells in the CNS or epidermis, as well as embryos with developmental patterning defects (Figure 2, D and E).

Isogenized *cn bw sp* homozygous males were fed 35 mM ethyl methanesulfonate (EMS) and crossed to females heterozygous for a dominant temperature-sensitive mutation (*DTS91 pr cn*) and a *CyO* balancer with a *lacZ* gene under the control of the *Ubx* promoter. Single balanced isolines were established by selecting against the *DTS91* chromosome at 29° as detailed in Royzman *et al.* (1997). The third chromosome lines analyzed were established in the laboratory of Ruth Lehmann (Skirball Institute) (Moore *et al.* 1998). These also were EMS mutagenized by the use of an isogenized *ru st P[faf-lacZ] e^s^ ca* chromosome. The third chromosome lines were balanced over *TM3 Sb*, *P[Ubx-lacZ]*. The second chromosome lines were estimated to have 2.7 lethal mutations per chromosome and third chromosome lines 1.9.

For screening, 8- to 15-hr embryos were collected from each mutant line, fixed, and hybridized to riboprobes for *lacZ* and *PCNA*. The *Ubx-lacZ* pattern (Figure 2C) distinguished heterozygous embryos with the balancers from the homozygous mutant embryos. The *faf-lacZ* reporter is expressed in the germ cells and did not interfere with analysis of the *PCNA in situ* pattern. The *in situ* hybridization was performed with multiwell baskets to analyze 144 lines at a time, as described (Royzman *et al.* 1997).

### Phenotypic analysis

The mutants producing embryos with putative postblastoderm division defects and large cells in the central nervous system (CNS) or epidermis initially were retested by DAPI staining (1 μg/mL) to visualize all embryonic nuclei. Those with apparent cell-cycle defects were characterized further by the incorporation of 5-bromo-2-deoxyuridine to examine S-phase (Smith and Orr-Weaver 1991) and by staining with antibodies to phospho-H3 and tubulin to analyze mitosis (Dej *et al.* 2004).

The thoracic *vs.* abdominal fate of the NB6-4 neuroblast in the *Cyclin E* mutants was determined by staining with antibodies for Eagle [rabbit antibody diluted 1:1000 (Higashijima *et al.* 1996)] and Repo [mouse antibody diluted 1:10 (DSHB)], as described by Akiyama-Oda *et al.* (2000) with modifications. Embryos were fixed in a mixture of 4% paraformaldehyde in 1× phosphate-buffered saline (PBS) with heptane (at 1:1 ratio) and then devitellinized with 100% methanol. After rehydration into 1× PBSTr (1X PBS plus 0.1% TritonX-100) the samples were blocked for 1 hr in blocking/antibody incubation buffer (1× PBSTr, 2% each of goat and donkey serum). After overnight incubation with primary antibody at 4°, the embryos were washed in 1× PBSTr then bound with antirabbit or antimouse secondary antibodies (1:500; Jackson ImmunoResearch) at room temperature for 1 hr. Stained embryos were mounted in Vectashield and examined with a Zeiss LSM 510 confocal system.

### Mutant mapping

The mutants were mapped by meiotic recombination with visible markers, male recombination (Chen *et al.* 1998), and by complementation tests using the Bloomington Chromosome *2* and *3* deficiency kits. Precise mapping of *cal1^2k32^* was done with small deletions generated by Parks *et al.* (2004). In complementation tests we first scored for lethality and then for the embryonic *PCNA in situ* phenotype (for *Cyclin E* alleles) or the DAPI embryonic phenotype. The *cyclin E^32-8^* and *tum^32a-20^* mutations were recovered on the same chromosome and were separated by meiotic recombination.

### Molecular characterization

The mutant alleles were sequenced from single embryo DNA preps (Strumpf and Volk 1998) by squashing the embryo in 25 μL of Gloor and Engel’s buffer (10 mM Tris, pH 8.2; 1 mM EDTA; 25 mM NaCl; 200 μg/mL proteinase K), followed by incubation at 37° for 30 min and at 95° for 2 min. Heterozygous embryos were distinguished from the homozygous mutants by the presence of detectable polymerase chain reaction (PCR) products from the *Ubx-lacZ* gene on the balancer chromosome (Whittaker *et al.* 2000). For each PCR, 1–2 μL of this lysate was used. Candidate genes were PCR amplified and the products sequenced.

## Results and Discussion

### Summary of screen

A total of 3010 EMS-mutagenized second chromosomes and 1000 EMS-mutagenized third chromosomes were screened for alterations in the *PCNA in situ* pattern in embryos (Park 2006; Royzman *et al.* 1997). Transcription of the *PCNA* gene is induced when the first G1 phase occurs in embryogenesis and occurs in a dynamic pattern coincident with the onset of S phase in the endocycle (Figure 1 and Figure 2) (Duronio and O’Farrell 1994).

A total of 201 mutants exhibited aberrant *PCNA* expression. For approximately 75% of these (148), this was likely a consequence of developmental abnormalities, as embryonic morphology was grossly disrupted. The remaining 53 mutants fell into several categories: (1) 15 reduced levels of *PCNA* transcripts. Seven of these were shown to be alleles of the E2F transcription factor subunits dDP and dE2F1 that promote *PCNA* transcription (Royzman *et al.* 1999; Royzman *et al.* 1997). (2) A total of 18 had defects in the postblastoderm divisions that were detectable in the *PCNA in situ* pattern (Figure 2D). Those mutations that were cloned or shown to correspond to known genes have been described in previous publications. We recovered four alleles of *dup* (Cdt1) (Whittaker *et al.* 2000), one of *cenp-C* (Heeger *et al.* 2005), two of the condensin subunit *dcap-g* (Dej *et al.* 2004), one of *thr* (a subunit of Drosophila Separase) (Dej *et al.* 2004), and two of *pim* (the Drosophila Securin) (Dej *et al.* 2004). (3) A total of 10 mutants had large cells in the central nervous system (Figure 2E). (4) A total of 10 mutants had persistent *PCNA* transcripts in the endocycle domains (Figure 2F). Here we focus on mutants from the last two categories that were molecularly characterized.

### New *cyclin E* alleles

Mutants were recovered whose *PCNA* transcripts failed to be down-regulated in the anterior and posterior midgut. Five of these mapped to the second chromosome, as does the *Cyclin E* gene, which when mutated causes persistent *PCNA* transcription in the endocycle domains (Figure 2F) (Duronio and O’Farrell 1995; Sauer *et al.* 1995). These five were shown to be alleles of *Cyclin E* by complementation tests with the *CycE^05206^* lethal allele, as well as *Df(2L)TE35D-1*, which deletes the *Cyclin E* gene. The putative alleles were lethal over both the deficiency and the *CycE^05206^* allele. The lethal phase suggested varying strength of the alleles, as *cyclin E^B29-25l^*, *cyclin E^22t^*, and *cyclin E^10.73^* were embryonic lethal over the deficiency, whereas *cyclin E^32-8^* and *cyclin E^1f36^* were larval lethal *in trans* to the deficiency. In addition to failure to complement for lethality, we confirmed that the transheterozygous embryos exhibited the mutant *PCNA in situ* phenotype. Moreover, the *cyclin E^B29-25l^*, *cyclin E^22t^*, and *cyclin E^32-8^* alleles were found to be viable with rough eyes and female sterile *in trans* to the *CycE^2^* female-sterile allele.

Four of the five new *Cyclin E* mutations were sequenced and the predicted protein changes identified (Table 1). The severity of the protein changes matched the phenotypic analysis, as the three strongest alleles are expected to cause truncated proteins (Table 1). The *cyclin E^1f36^* missense allele has been extensively characterized for its effects on follicle cell amplification during ovarian development (Park *et al.* 2007).

**Table 1:** New alleles of known cell-cycle genes

Gene	New Allele	Protein Change
*cyclin E*	*cyclin E^B29-25l^*	K24 changed to stop codon
	*cyclin E^22t^*	W349 changed to stop codon
	*cyclin E^10.73^*	Deletion of adenine at 1133, relative to the AUG codon, causes frameshift and stop codon at amino acid 407
	*cyclin E^1f36^*	G249 changed to E (Park *et al.* 2007)
	*cyclin E^32-8^*	Not sequenced; identified by complementation test
*pav*	*pav^3C53^*	Not sequenced; identified by complementation test
*tum (RacGAP50C)*	*tum^32a-20^*	L464 changed to H
*cal1*	*cal1^2k32^*	Q930 changed to stop codon
*spc105R*	*spc105R^IR8^*	T361 changed to M, K367 changed to stop codon, and V392 changed to M
	*spc105R^3C157^*	Not sequenced; identified by complementation test

### Neuroblast identity requires normal cyclin E function

**Figure 3:**
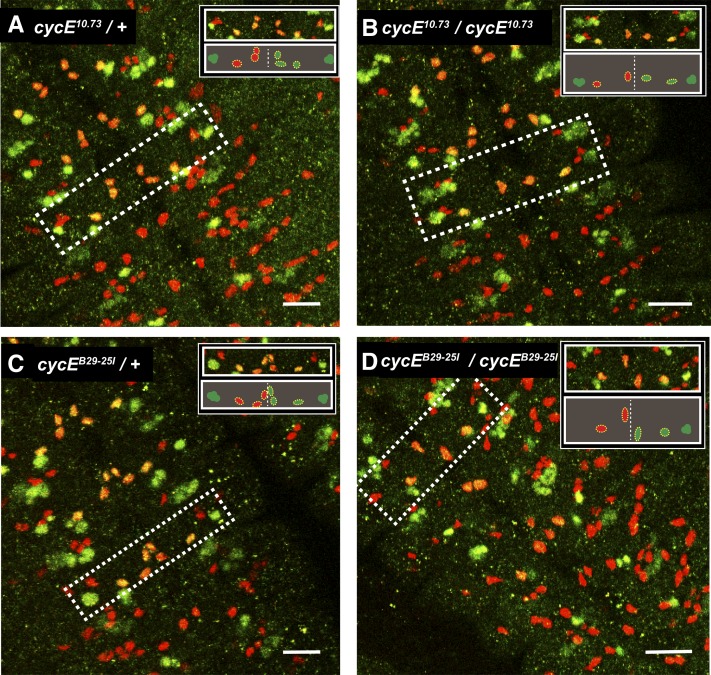
Neuroblast fate changes in *Cyclin E* mutants. The fate of neuroblast NB6-4t was scored by staining stage 15 embryos with antibodies against Eagle (green) and Repo (red). Eagle marks all cells in the NB6-4 lineage, and glia daughter cells stain also with Repo (and thus are yellow). The boundary between the T3 thoracic and A1 abdominal segments is visible by the absence of Eagle-labeled glia in the abdomen. In each panel the inset from the white box shows one thoracic segment stained (top) with an explanatory diagram (below). In the diagram the vertical dotted line marks the boundary of the two hemisegments. Cells from the NB6-4 lineage are indicated by yellow dotted lines. On the left the Repo-stained cells are shown; the right indicates the Eagle-stained cells. (A, C) In heterozygous controls NB6-4t in the T3 thoracic segment produces three glial progeny cells. In contrast, in both *Cyclin E* homozygous mutants (identified by the absence of the balancer *Ubx-lacZ* gene) (B, D), NB6-4t produces only two glial daughters, a fate normally seen for NB6-4a (Berger *et al.* 2005). T3 is highlighted for *cyclin E^10.73^* and T1 for *cyclin E^B29-25l^*. In the *cyclin E^10.73^* mutant in (B) the fate transformation is not present in all thoracic segments. See Table 2 for quantification. Scale bars, 20 μm.

In embryos homozygous mutant for the null allele of *Cyclin E*, *cyclin E^AR95^*, other workers found that the identity of a specific neuroblast was altered (Berger *et al.* 2005). In the thorax, NB6-4t divides asymmetrically to produce neurons and glia, whereas in the abdomen the corresponding neuroblast, NB6-4a, divides symmetrically to generate glia progeny. Strikingly, in the *Cyclin E* allele tested, NB6-4t divides symmetrically. This effect was proposed to be independent of the cell-cycle function of Cyclin E, as other cell-cycle regulators did not cause this transformation in neuroblast fate (Berger *et al.* 2005). We exploited the new alleles of *Cyclin E* to test whether loss of *Cyclin E* function consistently caused this change and whether the extent of neuroblast transformation would correlate with allele strength. We examined two of the new *Cyclin E* alleles that are predicted to cause truncated proteins forms and observed they also caused NB6-4t to be transformed to NB6-4a (Figure 3). In contrast, no fate transformation occurred in the weak *cyclin E^1f36^* allele (Table 2), indicating this allele retained sufficient Cyclin E function for neuroblast determination. The observation that all strong alleles of *Cyclin E* tested caused the same defect in neuroblast fate demonstrates that necessity of Cyclin E function, although it does not permit us to distinguish whether this is mediated by its cell-cycle function or an additional activity of Cyclin E.

**Table 2:** Neuroblast transformation in *cyclin E* mutants

Genotype	% Transformation NB6-4 from Thorax to Abdomen	Number Hemisegments Scored
*cycE^10.73^/+*	0	48
*cycE^10.73^/ cycE^10.73^*	93	30
*cycE^B29-25l^/+*	0	42
*cycE^B29-25l^ / cycE^B29-25l^*	94	36
*cycE^22t^/+*	0	72
*cycE^22t^/ cycE^22t^*	ND*^a^*	42
*cycE^1f36^/+*	0	42
*cycE^1f36^/ cycE^1f36^*	0	36

a These embryos did not develop to the stage to score the neuroblast transformation. ND, not determined.

### New alleles of cell division genes

**Figure 4:**
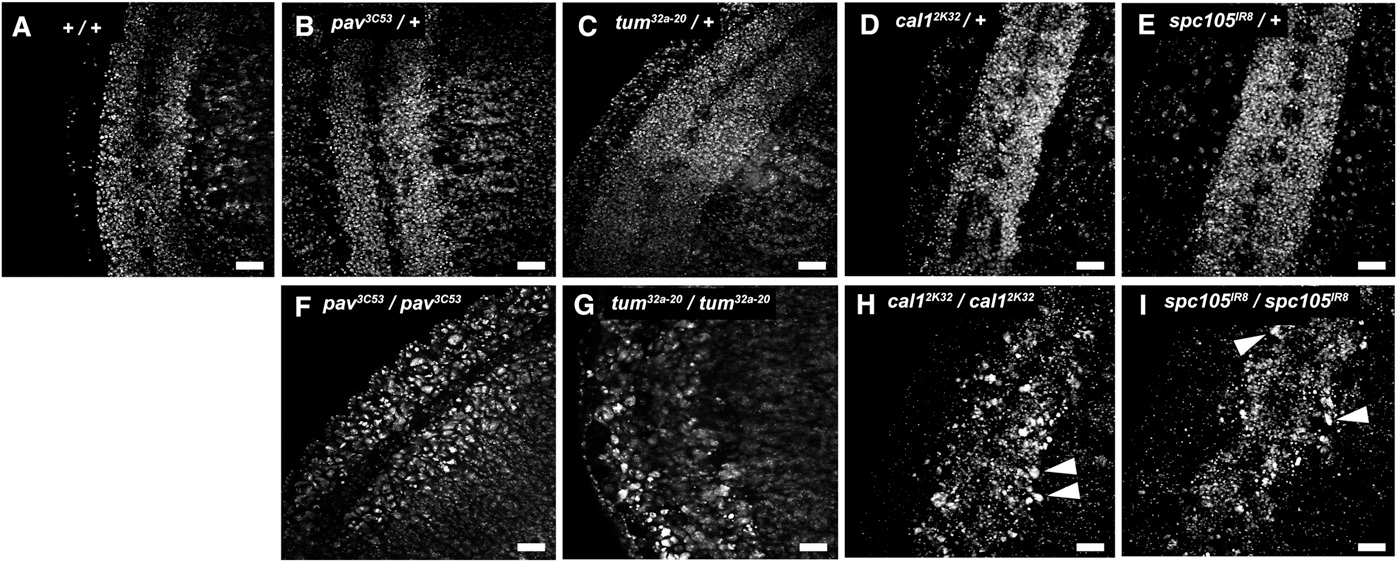
DAPI embryonic phenotypes. DAPI staining of the CNS and surrounding epidermis from stage 14 or 15 embryos. Homozygous embryos were distinguished from heterozygous controls by a *Ubx-lacZ* marker on the balancer chromosome. (A−E) Wild-type and heterozygous control embryos, stage 15. (F−I) Homozygous mutant embryos of designated genotype. DAPI staining of the embryos revealed that *pav^3C53^* and *tum^32a-20^* had cells with enlarged nuclei throughout the nerve cord and in the epidermis, whereas *cal1^2k32^* and *spc105R^IR8^* had isolated cells in the nervous system with increased nuclear size and ploidy (arrowheads). The *pav* and *tum* mutants arrest at stage 14; the *cal1* and *Spc105R* are stage 15. Scale bars 20μm.

We investigated five of the mutants exhibiting cells with large nuclei in the nervous system or epidermis (Table 1). Two of these had cells with large nuclei throughout the CNS and in the epidermis, whereas three had large nuclei in isolated cells in the CNS (Figure 4). Mutations *IR8* and *3C157* were found to be in the same complementation group. These five mutants were isogenized and mapped.

Mutant *3C53* was mapped to the interval *63E1-2;64B17* by failure of *Df(3L)GN50* to complement the large nuclei phenotype. Because the cytokinesis gene *pavarotti (pav)* is in this interval, we tested *pav^B200^* and found that this also failed to complement (Somers and Saint 2003). In addition, we observed multinucleate cells in the CNS of *3C53* mutant embryos, consistent with cytokinesis defects. Thus, we conclude that *3C53* is an allele of the *pav* gene, which encodes a kinesin motor protein.

Mutant *32a-20* also exhibited defects consistent with cytokinesis failure. The mutant was uncovered by *Df(2R)CX1* and delineated to a region of 10 genes by male recombination. One of these genes is *tumbleweed* (*tum*), encoding RacGAP50C, a Rho family GTPase required for cytokinesis (Zavortink *et al.* 2005). Indeed, sequencing revealed that the mutation caused a Leu to His substitution in a conserved residue in the GAP catalytic domain box II (Table 1).

The three remaining mutations were found to affect centromere and kinetochore proteins (Table 1). Mutant *2k32* was mapped to the region *89E11-F1* by its failure to complement *Df(3R)Exel6176*. Sequencing of candidate genes in the interval revealed a stop codon in the *cal1* gene encoding a centromere protein (Erhardt *et al.* 2008; Goshima *et al.* 2007). This *2k32* mutant was confirmed to be a consequence of the *cal1* mutation, as the mutant phenotype is complemented by a *cal1* transgene (Y. Unhavaithaya and T. L. Orr-Weaver, unpublished data). The *IR8*, *3C157* complementation group was mapped to *77E2-78A4* by the observation that the alleles over *Df(3L)ri-XT1* caused lethality and large nuclei in the CNS. A mutation generating a stop codon was identified in the coding sequence for the kinetochore protein SPC105R in the *IR8* mutant (Schittenhelm *et al.* 2009). Thus, we conclude that *IR8* and *3C157* are alleles of *Spc105R* (Table 1).

This study identified new alleles of known cell-cycle regulators that affect embryogenesis. These include alleles of *Cyclin E* with a range of severity, and the strongest cause transformation of the cell fate of the neuroblast NB6-4. It is striking that cytokinesis functions and centromere/kinetochore proteins display embryonic lethality when disrupted. One possibility is that aspects of cell division in the nervous system make it particularly vulnerable to mitotic defects. A simpler explanation, however, is that the continued mitotic divisions that occur in the nervous system after the rest of the embryonic cells have exited the cell cycle or entered the endocycle deplete remaining maternal pools to reveal defects in the zygotic mutants.

The three *PCNA in situ* phenotypes linked to defects in the cell cycle or division were readily scored: arrest in the postblastoderm divisions, the presence of large cells in the nervous system, and persistence of *PCNA* transcripts in the endocycle domains. Thus it is notable that a small number of mutants were identified. The second chromosome screen was more exhaustive than the third, corresponding with the recovery of multiple alleles in many of the genes identified (five in *Dp*, four in *dup*, five in *Cyclin E*). Eight postblastoderm arrest mutants, which were not cloned, are all single-allele complementation groups. We conclude that although our screen was not saturating, a limited number of genes essential for cell division can mutate to cause embryonic defects. This most likely is explained by persistence of maternal pools through the end of embryogenesis. The alleles presented here will be a valuable resource for analysis of the mechanisms and regulation of cell division in development.
